# Supplementary material for: High-versus conventional-volume pericapsular nerve group (PENG) block for total hip arthroplasty: A randomized, controlled trial
Source: PLoS One. 2026 Apr 17;21(4):e0343615. doi: 10.1371/journal.pone.0343615 (PMC13089707; doi:10.1371/journal.pone.0343615)
Supplement: S2 File — (DOCX) [file pone.0343615.s002.docx]

**Protocol**

**Research protocol: part 1**

**Project summary and background information**

Total hip arthroplasty(THA) continues to increase globally[1-3]. However, Patients undergoing THA usually experience a moderate or even severe postoperative pain[4-7]. Common regional blocks such as lumbar plexus block, fascia iliaca block and femoral nerve block often weaken the quadriceps femoris, or the analgesic effect is questioned. A new regional block，pericapsular nerve group (PENG) block, can significantly reduce the resting and dynamic pain score (NRS score) of patients with hip fracture, and preserve the motor function of the affected limb[8]。However, the volume of local anesthetics usually used by anesthesiologists to perform PENG block is 20 ml, which is called high volume when the volume rises above 30 ml. The high volume PENG block seems to show the characteristics similar to lumbar plexus block[9, 10]. Therefore, we speculate that with the increase of volume, the analgesic effect of PENG nerve block will be further improved, but there may be more patients with lower limb motor block. If high-volumevolume PENG block can significantly improve the analgesic effect after THA, but quadriceps weakness does not increase greatly, high-volume PENG block still has certain clinical application value. Up to now, the reports of high volume PENG block are mostly limited to case studies. We intend to a randomized, controlled trial to compare the effects of high- versus conventional-volume PENG blocks on postoperative pain and rehabilitation in patients undergoing THA, and explore the optimal volume of PENG block for THA. Our main purpose is to verify that high volume PENG block has better analgesic effect than conventional volume PENG block, but there may be more quadriceps weakness.

Expected outcomes: Compared with conventional volume PENG block, high volume PENG block has better analgesic effect, but it may have a higher incidence of quadriceps weakness.

**General information**

Protocol title:

**High- versus conventional-volume pericapsular nerve group (PENG) block for total hip arthroplasty: A randomized, controlled trial**

Date: This study was conducted between August 2024 and December 2024.

### Experimenters and Project management

Qiansong Wang; Third Affiliated Hospital of Chongqing Medical University (Gener Hospital), No.1 Shuanghu Branch Road, Chongqing, 401120, [650667@hospital.cqmu.edu.cn](mailto:650667@hospital.cqmu.edu.cn)

Yang Zhao; Affiliated Hospital of North Sichuan Medical College, No. 1 The South of Maoyuan Road, Nanchong, 637000, [594624370@qq.com](mailto:594624370@qq.com);

Xia Yuan, Third Affiliated Hospital of Chongqing Medical University (Gener Hospital), No.1 Shuanghu Branch Road, Chongqing, 401120, [405084258@qq.com](mailto:405084258@qq.com)

Zhao-Hui Chen, Third Affiliated Hospital of Chongqing Medical University (Gener Hospital), No.1 Shuanghu Branch Road, Chongqing, 401120, 650688@cqmu.edu.cn

Shi-Ming Qin; Third Affiliated Hospital of Chongqing Medical University (Gener Hospital), No.1 Shuanghu Branch Road, Chongqing, 401120, [650216@hospital.cqmu.edu.cn](mailto:650216@hospital.cqmu.edu.cn)

Chong-Mei Gao, Third Affiliated Hospital of Chongqing Medical University (Gener Hospital), No.1 Shuanghu Branch Road, Chongqing, 401120, [650094@cqmu.edu.cn](mailto:650094@cqmu.edu.cn)

ZC, QW, YZ and XY participated in and designed all of the experiments. CG,QW and SQ helped study conduct and data collection. QW and ZC analyzed the data and wrote the manuscript. ZC supervised the experiments and is the guarantor of the study.

**Study goals and objectives**

Our primary objective is to determine whether high volume PENG block has better analgesic effect than conventional volume PENG block, but it may have a higher incidence of quadriceps weakness, and explore the optimal volume of PENG block for THA.

**Study design**

Study Type: A randomized, controlled trial.

Study population:

Inclusion criteria: (1) Patients aged ≥18 years old; (2) those classified as American Society of Anesthesiologists physical status (ASA) grades 1-3; (3) those with a body weight of 45-90 kg; (4) those receiving primary unilateral THA, a posterior lateral approach; (5) those with no contraindications to regional or intravertebral anesthesia; and (6) those who could reliably report symptoms to the researchers.

Exclusion criteria: (1) Patient refusal; (2) those with a history of local anesthetic allergy; (3) those with infection near the puncture site: (4) those with dementia or cognitive impairment; (5) those with moderate or severe anemia; (6) pregnant patients; (7) those with chronic pain; and (8) those with long-term intake of painkillers such as opioid.

Methodology

A nurse anesthetist who was not involved in the study randomly divided patients into two groups using a random number table in a 1:1 ratio: the high-volume group (even numbers) and the conventional-volume group (odd numbers). The grouping data were sealed in sequentially numbered opaque envelopes, which were opened on the day of surgery by researchers who performed the nerve block. All nerve blocks were performed by a trained anesthesiologist who was proficient in PENG blocks. Another anesthesiologist from the acute pain service team who was not involved in the nerve block procedure or intraoperative management and was blinded to the grouping of the patients followed up with the patients after surgery to assess postoperative pain scores and sensory and motor blocks and collect data. The unblinding was performed after the completion of patient data collection.

The ultrasound-guided PENG block was performed preoperatively, with routine monitoring of blood pressure, oxygen saturation, and electrocardiogram; the patient was placed in the supine position. The ultrasound convex array probe (2-5 Hz) was placed on the inner and caudal sides of the anterosuperior iliac spine to identify the anteroinferior iliac spine, iliopubic eminence, and iliopsoas tendon. Using an in-plane technique and a lateral to medial orientation, the block needle was advanced until its tip was located on the periosteum on the dorsal side of the iliopsoas tendon. After negative aspiration, the high-volume group was injected with 40 mL of 0.375% ropivacaine, and the conventional-volume group received 20 mL of 0.375% ropivacaine, resulting in a local anesthetic between the periosteum of the iliopsoas tendon and iliopubic eminence. Dexamethasone(5mg) was mixed with the local anesthetic and administered simultaneously in each group.

After completion of the nerve block, a spinal anesthesia puncture was performed immediately in the lateral position at the L3-4 interspace. Subsequently, 10 mg of bupivacaine (volume = 2 mL) was injected, Both groups were given a patient-controlled analgesia electronic pump (no continuous background dose, 0.16 mg of hydromorphone bolus; lockout interval = 10 min) at the end of the operation.

#### The primary outcomes were the dynamic pain scores (with hip adduction) reported by patients using a 0-10 VAS (0 indicated no pain and 10 indicated worst pain imaginable) at 6 h post-surgery. Secondary outcomes included static pain scores (at rest) at 3 h, 6 h, 24 h, and 48 h post-surgery, dynamic pain scores of patients at 3 h, 24 h, and 48 h post-surgery,sensory and motor block of lower limbs at 3 h, 6 h, and 24 h post-surgery. Other secondary outcomes included the time of first opioid consumption (time of first analgesia pump press), total opioid consumption within 48 h, opioid-related complications (e.g., nausea, vomiting, dizziness, pruritus, and respiratory depression), time of first walking, length of hospital stay, falls during hospitalization, post-operative infection, local anesthetic poisoning during nerve block, and vascular puncture.

#### Post-operative sensory block was evaluated in the anterior, lateral and medial aspects of the mid-thigh innervated by the femoral nerve, lateral femoral cutaneous nerve, obturator and femoral nerve respectively at 3 h, 6 h, and 24 h post-surgery using the Aliste J's method. For each region, the blockade was evaluated on a 3-point scale: 2 = no block, 1 = analgesia (the patient can feel touch, not cold), 0 = anesthesia (patient can't feel touch). Post-operative motor block was investigated through knee extension, which was examined while in supine position with the patient’s hip and knee flexed at 45° and 90°, respectively. The patient was asked to extend the knee against gravity and resistance, the extension was graded based on a 3- point scale: 2=no block (extension against gravity and against resistance); 1= paresis (extension against gravity but not against resistance); 0=paralysis (no extension possible). First walking was defined as the ability to take at least three steps for the first time post-operatively using a walker. Post-operative consumption of intravenous hydromorphone was converted into intravenous morphine equivalent at the ratio of 1.5: 10.

**Safety considerations**

**1**、 Anesthesiologists are keeping monitoring the vital signs of the patients during the whole period.

2、To avoid local toxicity syndrome occurring, the patient's minimum weight should not be less than 45kg. Before administering local anesthetic, negative aspiration should be applied to ensure that local anesthetic does not enter the blood.

3、If the local toxicity syndrome occurred, midazolam or propofol would be administered intravenously for sedation, also positive mask oxygenation was required, and trachea intubation, fluid therapy, adrenaline therapy, or cardiopulmonary resuscitation would be performed if needed.

**Follow-up**

Follow up until each patient's discharge with no adverse events.

**Data management and statistical**

The collected data is kept and locked by an anesthesiologist**.**SPSS 20.0 software was used for statistical analyses. The normality of continuous data was assessed using the Shapiro-Wilk test. For normally distributed continuous data, results are presented as mean ± standard deviation (SD) and compared using a two-sample independent t-test. Non-normally distributed continuous variables are expressed as median and interquartile range [IQR] (M [P25, P75]) and compared using the Mann-Whitney U test. Categorical data were expressed as percentages and compared using the χ² test or Fisher’s exact test as appropriate. A two-sided P<0.05 was considered statistically significant. The log-rank test was used to evaluate time-to-event data.

**Problems anticipated**

**None.**

**Ethics**

This study was approved by the Medical Ethics Committee of the third affiliated hospital of Chongqing Medical University (president Fei Hao), China on 16 July 2023, approval number- 2023/27. The study protocol was registered with the Chinese registry of clinical trials (http://www.chictr.org.cn) (**ChiCTR2300077281**; November 3, 2023) and conducted in accordance with the Helsinki Declaration-2013.

**Informed consent forms**

The participants provided written consent. All methods were carried out in accordance with the Declaration of Helsinki.

**Budget**

None.

### Other support for the project

None.

### Other research activities of the investigators

None.

### Financing and insurance

None.

### References

**1.** Matsuoka H, Nanmo H, Nojiri S, Nagao M, Nishizaki YJJoosojotJOA. Projected numbers of knee and hip arthroplasties up to the year 2030 in Japan. 2023; 28(1): 161-6. <https://doi.org/10.1016/j.jos.2021.09.002> PMID: 34593285

**2.** Ahmed H, Al-Dadah OJAoB. Total Hip Arthroplasty in fracture neck of femur: A review of the literature. 2023; 89(1): 29-36. <https://doi.org/10.52628/89.1.8497> PMID: 37294982

**3.** Zeelenberg ML, Den Hartog D, Panneman MJM, Polinder S, Verhofstad MHJ, Van Lieshout EMM. Trends in incidence, health care consumption, and costs for proximal femoral fractures in the Netherlands between 2000 and 2019: a nationwide study. Osteoporos Int. 2023; 34(8): 1389-99. <https://doi.org/10.1007/s00198-023-06774-y> PMID: 37119329

**4.** Bober K, Kadado A, Charters M, Ayoola A, North T. Pain Control After Total Hip Arthroplasty: A Randomized Controlled Trial Determining Efficacy of Fascia Iliaca Compartment Blocks in the Immediate Postoperative Period. J Arthroplasty. 2020; 35(6): S241-S5. <https://doi.org/10.1016/j.arth.2020.02.020> PMID: 32222267

**5.** Tay HP, Wang X, Narayan SW, Penm J, Patanwala AE. Persistent postoperative opioid use after total hip or knee arthroplasty: A systematic review and meta-analysis. Am J Health Syst Pharm. 2022; 79(3): 147-64. <https://doi.org/10.1093/ajhp/zxab367> PMID: 34537828

**6.** Højer Karlsen AP, Geisler A, Petersen PL, Mathiesen O, Dahl JB. Postoperative pain treatment after total hip arthroplasty. Pain. 2015; 156(1): 8-30. <https://doi.org/10.1016/j.pain.0000000000000003> PMID: 25599296

**7.** Zheng J, Pan D, Zheng B, Ruan X. Preoperative pericapsular nerve group (PENG) block for total hip arthroplasty: a randomized, placebo-controlled trial. Reg Anesth Pain Med. 2022; 47(3): 155-60. <https://doi.org/10.1136/rapm-2021-103228> PMID: 34873023

**8.** Giron-Arango L, Peng PWH, Chin KJ, Brull R, Perlas A. Pericapsular Nerve Group (PENG) Block for Hip Fracture. Reg Anesth Pain Med. 2018; 43(8): 859-63. <https://doi.org/10.1097/AAP.0000000000000847> PMID: 30063657

**9.** Ahiskalioglu A, Aydin ME, Ozkaya F, Ahiskalioglu EO, Adanur S. A novel indication of Pericapsular Nerve Group (PENG) block: Prevention of adductor muscle spasm. J Clin Anesth. 2020; 60: 51-2. <https://doi.org/10.1016/j.jclinane.2019.08.034> PMID: 31445179

**10.** Ahiskalioglu A, Aydin ME, Celik M, Ahiskalioglu EO, Tulgar S. Can high volume pericapsular nerve group (PENG) block act as a lumbar plexus block? J Clin Anesth. 2020; 61: 109650. <https://doi.org/10.1016/j.jclinane.2019.109650> PMID: 31732426
