# Supplementary material for: High-versus conventional-volume pericapsular nerve group (PENG) block for total hip arthroplasty: A randomized, controlled trial
Source: PLoS One. 2026 Apr 17;21(4):e0343615. doi: 10.1371/journal.pone.0343615 (PMC13089707; doi:10.1371/journal.pone.0343615)
Supplement: Attachment — Submitted filename: Response to Reviewers .docx [file pone.0343615.s004.docx]

Manuscript No.: PONE-D-25-27762

Title: High-versus conventional-volume pericapsular nerve group (PENG) block for total hip arthroplasty: A randomized, controlled trial

Dear Prof Rizaldy Taslim Pinzon,

Thank you very much for your letter and helpful advice. We have revised the manuscript and would like to re-submit it for your consideration. We would like to express our sincere thanks to you and the reviewers for your constructive and positive comments which help us promote our manuscript a lot.

We have addressed all the comments raised by the editor and reviewers, and the amendments are highlighted in red in the revised manuscript. Besides, we checked the format according to your journal submission guidelines. Also, we have listed a point-by-point response to the editor and reviewers' comments as follows.

Your consideration of our revised manuscript for publication in your journal will be highly appreciated!

We look forward to hearing from you soon.

Best regards,

Zhao-Hui Chen,

Department of Anesthesiology,

Third Affiliated Hospital of Chongqing Medical University ,

 No.1 Shuanghu Branch Road,

Chongqing, 401120, China;

Email: 650688@cqmu.edu.cn

**Response to the reviewer 1**

**Manuscript No.:** PONE-D-25-27762

**Title**: High-versus conventional-volume pericapsular nerve group (PENG) block for total hip arthroplasty: A randomized, controlled trial

**Comment 1:** Line 114: second was needs to be deleted.

**Response:** Thank you for your helpful and valuable comment. We have deleted unnecessary and lengthy expressions in brackets（line124-125）. Revision details were shown below

Clinical Trial Registry (~~ChiCTR,~~ https://www.chictr.org.cn/~~, ID:~~ ;**ChiCTR2300077281;**~~, Date of registration:~~November 3, 2023) before patient recruitment.

**Comment 2:** Line 224: Table 2; P value P should be italic.

**Response:** Thank you very much for your kind reminding. We have changed the *P* value in all tables to italics. Please check them in Table2-4 of the revised manuscript.

**Comment 3:** P-values up to 2 decimal points are sufficient.

**Response**: Thank you for your helpful and valuable comment. we have revised all *P* values with only 2 decimal points throughout the manuscript.

**Comment 4:** Multiple testing adjustment will make the secondary end points more non-significant and hence no need.

**Response**: Thank you for your helpful and valuable comment. We deleted the dynamic and static VAS pain scores at 48 hours after surgery. Please check them in Table2 of the revised manuscript. Related descriptions have also been removed.(line59,line115,line180-181)

**Comment 5:** Table 4. time to first opioid hazards ratio seems to be 9.5 vs 5.3 but not significantly different. Please check this result. Although it is not significant; is the difference in right direction which needs to be commented.

**Response**: Thank you for your helpful and valuable comment. We checked the original data and statistical process, and have supplemented the discussion of these results, and the details are shown below(Discussion section, line 318-328).

There was no significant difference between the two groups in time to first opioid use or in the proportion of patients who did not require opioids within 48 hours after operation. However, the conventional volume group showed a trend toward a later time to the first opioid use and a higher proportion of patients without opioid use. The possible reason was that although there was no statistically significant difference between the groups in preoperative pain scores or the proportion of patients not requiring opioids. the average preoperative dynamic pain score and the proportion of preoperative opioid use were higher in the high-volume group. The high-volume PENG block failed to reverse the preoperative pain distribution in THA patients after operation. Furthermore, as our sample size reached the pre-specified target, the likelihood of a type II error in our conclusion is very low, which further supports that the postoperative analgesic effect of high-volume PENG block is not superior to that of the conventional volume group.

**Response to the reviewer 2**

**Manuscript No.:** PONE-D-25-27762

**Title**: High-versus conventional-volume pericapsular nerve group (PENG) block for total hip arthroplasty: A randomized, controlled trial

**Comment 1:** The results and discussion sections lack concise structuring. The narrative is repetitive in places, particularly where similar findings are discussed across multiple studies (e.g., Ahiskalioglu et al. and Girón-Arango et al.). Consider synthesizing this information to avoid redundancy and focus on the novel contribution of your study.

**Response:**Thank you for your helpful and valuable comment. We have greatly revised the results （line233-242,line247-258）and discussions section (line263-271,line272-294)., trying to be concise, particularly where similar findings are discussed across multiple studies (e.g., Ahiskalioglu et al. and Girón-Arango et al.)。 Details were shown below:

The dynamic pain scores of the high- and conventional-volume groups at 6 h after surgery were 5.0(4.0,6.0) and 4.0(4.0,5.0), respectively, indicating that their dynamic pain scores showed no statistically significant differences (Table 2). ~~No significant differences in the dynamic pain scores were found at 3 h and 24 h between the two groups. Meanwhile, no statistically significant differences in the static pain scores were observed between the two groups during all study time points (Table 2). There was no significant difference in the dermatomal sensory block of the lateral, anterior and medial thigh on the surgical side between the two groups. Similarly, no significant differences were observed in the motor block on the surgical side between the two groups (Table 3).~~ There was no significant differences were observed between the two groups in dynamic pain scores at 3 and 24 hours, static pain scores at any time point, dermatomal sensory block, or motor block on the surgical side. (Table 2,3).

Four patients (21.1%) in the conventional-volume group and one patient (5.6%) in the high-volume group did not require any opioids for 48 h. ~~No statistically significant differences in the proportion of opioid requirements, time of primary opioid consumption, and total opioid consumption within 48 h were found between the two groups (Table 4). No statistically significant differences in the time of first walking and length of hospital stay were observed between the two groups. No statistically significant differences in the incidence of post-operative opioid-related complications (e.g., nausea, vomiting, pruritus, and dizziness) were found between the two groups (Table 4).~~ There was no significant differences in the proportion of opioid requirements, time of primary opioid consumption, total opioid consumption within 48 h, time of first walking , length of hospital stay and incidence of post-operative opioid-related complications were found between the two groups (Table 4). Post-operative respiratory depression, in-hospital falls, wound infections, intravascular injections, and local anesthetic poisoning were observed in neither group during nerve block (Table 4).

In this randomized trial, we compared high-volume versus conventional-volume PENG blocks in patients undergoing primary THA. ~~The results revealed no statistically significant differences in the dynamic pain scores between the two groups at 6 h post-operatively.~~ At 6 h post-operatively, the two groups showed no statistically significant difference in dynamic pain scores. ~~Similarly, no statistically significant differences in the dynamic pain score at other time-points and the static pain scores at all time-points were found.~~ Furthermore, dynamic pain scores at other time points and static pain scores throughout the entire period were statistically similar between the groups. In addition, ~~no statistically significant differences~~ there were no significant differences in the dermatomal sensory block and motor block were found at all time-points post-operatively.

Ahiskalioglu *et al.* found that the high-volume PENG block showed the characteristics of a lumbar plexus block ~~and the innervation areas of the obturator nerve, the femoral nerve, and the lateral femoral cutaneous nerve were sufficiently anesthetized.~~ [13, 18]. Given that long-acting local anesthetics used in nerve blocks provide post-operative analgesia lasting over 10 h, the pain scores within ~~the first 0–~~6 h post-operatively are expected to be significantly lower with high-volume PENG blocks compared to conventional-volume PENG blocks, which was inconsistent with the results of the present study. ~~However, it should be noted that quadriceps weakness occurs more frequently with high-volume PENG blocks. Nevertheless, the results of the present study are inconsistent~~ ~~with the trend reported by Ahiskalioglu~~ *~~et al~~*. In the study by Ahiskalioglu *et al*, during the application of PENG block, the needle tip was located on the medial side of the iliopsoas tendon, and the local anesthetic spread along the psoas major and pubic muscles, thus blocking the obturator nerve, the lateral femoral cutaneous nerve and the femoral nerve. Therefore, the lumbar plexus block-like characteristics ~~of the high-volume PENG block observed by Ahiskalioglu et al.~~ may be related to suboptimal PENG block ~~due to the aforementioned reasons~~. Elsewhere, Girón-Arango *et al*[15, 19]~~,~~ inferred that another reason ~~for the unexpected quadriceps myasthenia or the appearance similar to the lumbar plexus block in the PENG block~~ was that the local anesthetic injection was too superficial. In this study, ~~the PENG block was performed under intravenous analgesia and sedation, and~~ subcutaneous local anesthetic injections were contraindicated. The tip of the needle was required to reach the bone surface of the iliopubic eminence and stick to the lateral side of the iliopsoas tendon before injecting local anesthetic. ~~to avoid accidental superficial injection and unexpected diffusion of local anesthetic.~~ We found that the standard PENG block did not block the peripheral branches of lumbar plexus even with high volume ~~Our results confirm that high-volume PENG block does not block the obturator nerve effectively and will not increase the analgesic effect~~.

**Comment 2:** The methods describing the PENG block procedure lack detail regarding ultrasound guidance, needle type, and exact positioning. These are essential for reproducibility. Additionally, specify the criteria for evaluating motor and sensory block (e.g., scale used, evaluator blinding, etc.)

**Response:**Thank you for your helpful and valuable comment.

1.In the part of materials and methods, we described the whole procedure of ultrasound-guided PENG block, including the types and positioning methods of needles（Line151-162）and added a figure（figure 1） of ultrasound-guided PENG block. Please check it.Details were shown below:

**Performance of PENG blocks**

The ultrasound-guided PENG block was performed preoperatively, with routine monitoring of blood pressure, oxygen saturation, and electrocardiogram; the patient was placed in the supine position. The ultrasound convex array probe (2-5 Hz) (HITACHI ALOKA ARIETTA, Fujifilm Medical, Tokyo, Japan) was positioned on transverse orientation, medial and caudal to the anterosuperior iliac spine to identify the anteroinferior iliac spine, the iliopubic eminence and the psoas tendon. Using an in-plane technique and a lateral to medial orientation, the block needle (B. Braun Melsungen AG, Melsungen ，Germany) was advanced until its tip was located on the periosteum on the dorsal side of the iliopsoas tendon. After negative aspiration, the high-volume group was injected with 40 mL of 0.375% ropivacaine, and the conventional-volume group received 20 mL of 0.375% ropivacaine, resulting in a local anesthetic between the periosteum of the iliopsoas tendon and iliopubic eminence(Figure 1). Dexamethasone (5mg) was mixed with the local anesthetic and administered simultaneously in each group.

2.In the materials and methods section, we introduced the methods of evaluating motor and sensory block in detail(line187-195). Details were shown below:

Post-operative sensory block was evaluated in the anterior, lateral and medial aspects of the mid-thigh innervated by the femoral nerve, lateral femoral cutaneous nerve, obturator and femoral nerve respectively at 3 h, 6 h, and 24 h post-surgery using the Aliste J's method. For each region, the blockade was evaluated on a 3-point scale: 2 = no block, 1 = analgesia (the patient can feel touch, not cold), 0 = anesthesia (patient can't feel touch). Post-operative motor block was investigated through knee extension, which was examined while in supine position with the patient’s hip and knee flexed at 45° and 90°, respectively. The patient was asked to extend the knee against gravity and resistance, the extension was graded based on a 3- point scale: 2=no block (extension against gravity and against resistance); 1= paresis (extension against gravity but not against resistance); 0=paralysis (no extension possible).

3. In the materials and methods , we introduced the blind method of postoperative sensory and motor block evaluation in the materials and methods(line141-150). Details were shown below:

A nurse anesthetist who was not involved in the study randomly divided patients into two groups with approximately equal sample sizes using a random number table in a 1:1 ratio: the high-volume group (even numbers) and the conventional-volume group (odd numbers). The grouping data were sealed in sequentially numbered opaque envelopes, which were opened on the day of surgery by researchers who performed the nerve block. All nerve blocks were performed by a trained anesthesiologist who was proficient in PENG blocks. Another anesthesiologist from the acute pain service team who was not involved in the nerve block procedure or intraoperative management and was blinded to the grouping of the patients followed up with the patients after surgery to assess post-operative pain scores and sensory and motor blocks and collect data. Unblinding was performed once the data collection process was completed for all patients.

**Comment 3:** Sample size: State the software or method used for the power analysis (e.g., G*Power); Clearly specify the calculated sample size per group and total (e.g., 17 per group, 34 total);Justify the chosen minimum clinically important difference (MCID) clearly, noting it as a conservative estimate between moderate and severe pain.

**Response:** Thank you for your kind reminder and comment. In the part of sample size estimation, we added the name of the software used for sample size estimation. At the same time, we described why we chose 1.5 as the minimum dynamic pain score reduction value with clinical significance, and described how to calculate the sample size of each group and the total sample size(line200-212). Details were shown below:

Sample size calculation was performed using PASS version 11(Power Analysis and Sample Size Software).The minimum ~~decrease~~ clinically important difference in pain scores ~~that is considered meaningful to patients is associated with their~~ was determined based on patients’ baseline pain levels. For individuals experiencing moderate pain, a change of 1.3 on the VAS is the threshold for minimum clinically significant pain relief. In contrast, for those with severe baseline pain, a reduction of 1.8 on the VAS is deemed clinically significant[16]. A recent clinical trial reported that the dynamic pain score at 6 h after surgery in patients with a PENG block was 6[11], which was considered between moderate and severe pain. Therefore, we chose 1.5 as the clinically meaningful minimum dynamic pain score reduction for patients receiving the PENG block. Based on previous data on the use of the PENG block in THA[9, 17], the standard deviation (SD) was estimated to be 1.5. To test the difference in pain score of 1.5 (out of 10) and SD of 1.5, a two-tailed, independent samples t-test with an α-error of 0.05 and a β-error of 0.2 was conducted; a minimum of 17 patients were required in each group. Considering the imbalance of grouping and drop-outs, 40 patients were recruited.

**Comment 4:** The conclusion that high-volume PENG block is not superior could be strengthened by discussing whether a type II error is possible due to sample size or variability in clinical performance. Some caution in the interpretation would increase credibility. Moreover, the limitations are acknowledged but could be expanded. For example, the influence of spinal anesthesia on early motor/sensory assessments is important and should be emphasized further. Additionally, the use of subjective pain scores without standardized rescue analgesia criteria should be discussed.

**Response:** Thank you for your helpful and valuable comment.

1.We added the discussion on the influence of sample size and variability of clinical performance on the reliability of conclusions. Please check them out.(line318-328). Details were shown below:

There was no significant difference between the two groups in time to first opioid use or in the proportion of patients who did not require opioids within 48 hours after operation. However, the conventional volume group showed a trend toward a later time to the first opioid use and a higher proportion of patients without opioid use. The possible reason was that although there was no statistically significant difference between the groups in preoperative pain scores or the proportion of patients not requiring opioids. the average preoperative dynamic pain score and the proportion of preoperative opioid use were higher in the high-volume group. The high-volume PENG block failed to reverse the preoperative pain distribution in THA patients after operation. Furthermore, as our sample size reached the pre-specified target, the likelihood of a type II error in our conclusion is very low, which further supports that the postoperative analgesic effect of high-volume PENG block is not superior to that of the conventional volume group.

2. We rewrote the influence of spinal anesthesia on postoperative sensory and motor evaluation（line329-334）. Details were shown below:

Nonetheless, this study had several limitations. First, ~~spinal anesthesia was used in all patients, and~~ the dose of isobaric bupivacaine was 10 mg. Previous studies demonstrated that the median for acupuncture analgesia with 10 mg isobaric bupivacaine was 2 h (35-250 min) and the motor block median was 3 h (50-250 min)[23]. These data showed that spinal anesthesia ~~did not affect the overall assessment of motor and sensory block in the 0-6-h but had some effect on the evaluation of sensory motor block~~ would seriously affect the evaluation of sensorimotor block at 3 h post-operatively.

3.In terms of standardized rescue and analgesia after operation, in fact, we have done standardized rescue and analgesia measures, We We are sorry for missing this detail and added this content(line172-175). Details were shown below:

Both groups were given a patient-controlled analgesia electronic pump (no continuous background dose, 0.16 mg of hydromorphone bolus; lockout interval = 10 min) at the end of the operation and were trained for breakthrough pain(VAS pain scores≥4) in the ward.

**Comment 5:** Several grammatical issues and awkward phrases need editing.

**Response**: Thank you for your helpful and valuable comment. We invited a retouching organization to polish the full text carefullyext. Please check the details of these changes carefully(line51-54,line71-74,line84,line96-97,line103-104,line201-202,line222,line227,line346-347).

**Comment 6:** Ethical apporval? Registration of clinical trial?.

**Response**: Thank you for your helpful and valuable comment. We introduced ethical apporval and clinical trial registration in materials and methods(line122-129). Details were shown below:

This was a randomized, controlled trial was conducted at the Third Affiliated Hospital of Chongqing Medical University from August to November, 2024. This randomized trial was registered at the Chinese Clinical Trial Registry (~~ChiCTR,~~ https://www.chictr.org.cn/~~, ID:~~ ;**ChiCTR2300077281;**~~, Date of registration:~~November 3, 2023) before patient recruitment. The study was approved by the ethics committee of the third affiliated hospital of Chongqing Medical University and was conducted in accordance with the principles of the Declaration of Helsinki. The first patient was enrolled on August 29, 2024，and the last patient was followed up on November 30, 2024.The study protocol followed Consolidated Standards of Reporting Trials (CONSORT) guidelines.

**Comment 7:** Please ad this important ciation on PENG block: DOI:10.3390/jcm13092674

**Response**: Thank you for your helpful and valuable comment. We studied this article carefully, and cited it in the discussion(line309-311), please check it.
