# Supplementary material for: High-versus conventional-volume pericapsular nerve group (PENG) block for total hip arthroplasty: A randomized, controlled trial
Source: PLoS One. 2026 Apr 17;21(4):e0343615. doi: 10.1371/journal.pone.0343615 (PMC13089707; doi:10.1371/journal.pone.0343615)
Supplement: Attachment — Submitted filename: Response_to_Reviewers__auresp_2.docx [file pone.0343615.s005.docx]

Manuscript No.: PONE-D-25-27762R1

Title: High-versus conventional-volume pericapsular nerve group (PENG) block for total hip arthroplasty: A randomized, controlled trial

Dear Dr Nan Jiang,

Thank you very much for your letter and helpful advice. We have revised the manuscript and would like to re-submit it for your consideration. We would like to express our sincere thanks to you and the reviewers for your constructive and positive comments which help us promote our manuscript a lot.

We have addressed all the comments raised by the editor and reviewers, and the amendments are highlighted in red in the revised manuscript. Besides, We checked all the references and found that none of the cited references had been withdrawn.. Also, we have listed a point-by-point response to the editor and reviewers' comments as follows.

Your consideration of our revised manuscript for publication in your journal will be highly appreciated!

We look forward to hearing from you soon.

Best regards,

Zhao-Hui Chen,

Department of Anesthesiology,

Third Affiliated Hospital of Chongqing Medical University ,

 No.1 Shuanghu Branch Road,

Chongqing, 401120, China;

Email: 650688@cqmu.edu.cn

**Response to the reviewer**

**Manuscript No.:** PONE-D-25-27762R1

**Title**: High-versus conventional-volume pericapsular nerve group (PENG) block for total hip arthroplasty: A randomized, controlled trial

**Response to major points**

**Comment 1:**

Primary Outcome & Power Analysis

o The primary outcome (dynamic VAS at 6h) is clearly defined and analysed.

o The power calculation is now adequately justified, with an MCID of 1.5 points on the VAS and SD = 1.5. The use of PASS software is appropriate.

o However, the total analysed sample (n=37) is only marginally above the minimum of 34 required. Although dropouts were low, the risk of type II error cannot be entirely excluded. The authors argue that it is unlikely, but more cautious interpretation would be preferable.

**Response:** Thank you for your helpful and valuable comment. We have revised the description of type II errors,（line304-307）. Revision details were shown below:

Furthermore, ~~as~~ our sample size has reached the pre-specified target, ~~the likelihood of a type II error in our conclusion is very low,~~which reduces the probability of type II errors, which further supports that the postoperative analgesic effect of high-volume PENG block is not superior to that of the conventional volume group.

**Comment 2:**

Statistical Methods

o Analyses are appropriate: t-test/Mann-Whitney U for continuous variables, χ²/Fisher’s exact test for categorical data, log-rank test for time-to-event outcomes.

o P-values are now standardized to two decimal places. Confidence intervals are presented for time-to-event data but could be more consistently reported for other outcomes to improve interpretability.

o Adjustment for multiple testing was deliberately not performed, which is acceptable since secondary endpoints are exploratory, but this should be explicitly acknowledged in the Discussion to avoid misinterpretation.

**Response:** Thank you very much for your helpful and valuable comment. We increased the median difference and risk ratio and the 95% confidence interval. Please check them in Table2 and 4 of the revised manuscript. We have added the discussion of repeated measurement data(line319-320), revision details were shown below:

Additionally, some secondary endpoints involved repeated measurement data; however, adjustment for multiple testing was not performed since these endpoints were exploratory.

**Comment 3:**

Randomization & Blinding

o Randomization method (opaque envelopes with 1:1 allocation) is appropriate.

o Blinding is well described: block performers were not blinded, but assessors were. The potential for performance bias should be acknowledged more clearly.

**Response**: Thank you for your helpful and valuable comment. We have added a discussion about the limitations of blind method(line318-319), revision details were shown below:

Thirdly, in this study, assessors were blinded, while block performers were not, which may introduce potential performance bias.

**Comment 4:**

Outcome Assessments

o Sensory and motor block evaluation scales are now clearly described and reproducible.

o The influence of spinal anaesthesia on early block assessment is discussed, but the limitation remains important. At 3h post-op, residual spinal effect could confound sensory/motor findings. This undermines the reliability of early block outcome measures.

**Response**: Thank you for your helpful and valuable comment. Please check it in line 308-315 for the related limitation description

**Comment 5:**

Results & Interpretation

o No statistically significant differences were found between groups. The Discussion appropriately cites possible explanations (baseline imbalance in opioid use, volume-related spread inconsistencies).

o However, the interpretation could still overstate certainty. While the trial is adequately powered for the primary endpoint, the sample size is small for detecting uncommon complications or subtle functional differences.

**Response**: Thank you for your helpful and valuable comment. In the discussion of limitations,we added a description of the influence of small sample size on secondary results,and the details are shown below(Discussion section, line 321-322).

Fourthly, although the sample size was adequate for the primary endpoint, it was relatively small and may not have been sufficient to detect differences in the secondary endpoints.

**Comment 6:**

Transparency & Reporting

o CONSORT flow diagram and trial registration are now included.

o Ethical approval and informed consent are documented.

o Data availability statement is satisfactory.

o The manuscript is clearer after language editing, though minor stylistic inconsistencies remain.

**Response**: Thank you for your helpful and valuable comment. We invited a retouching organization to polish the full text carefully again. Please check the details of these changes carefully (line88-93,line104,106-111,line1139-146,line159-161,line232,line241,line244,line253-258,line260-270,line295-303)

**Response to** **minor Points**

**Comment 1:** Tables should consistently report both effect size estimates (mean/median difference) and 95% CI, not only P-values

**Response:**Thank you for your helpful and valuable comment. We added the median difference and 95% confidence interval in Table 2 and 4. Please check them

**Comment 2:** In Table 4, the trend toward later time-to-first opioid in the conventional group is acknowledged in the Discussion, but presenting hazard ratios with CI would better quantify this.

**Response:**Thank you for your helpful and valuable comment. We have added the risk ratio and 95% confidence interval in Table 4. Please check it.

**Comment 3:** The claim that a “type II error is very unlikely” should be softened to reflect uncertainty.

**Response:** Thank you for your kind reminder and comment. We have revised the relevant description(line304-7), and the details are as follows:

Furthermore, ~~as~~ our sample size has reached the pre-specified target, ~~the likelihood of a type II error in our conclusion is very low,~~which reduces the probability of type II errors, which further supports that the postoperative analgesic effect of high-volume PENG block is not superior to that of the conventional volume group.

**Comment 4:** The conclusion would be more balanced if it emphasized that conventional volume is sufficient, rather than definitively “optimal.”

**Response:** Thank you for your helpful and valuable comment. We have revised the relevant description(line327-330), and the details are as follows:

~~Consequently, the conventional volume appears to be the optimal dose of PENG block for~~ Therefore, the conventional volume is sufficient for PENG block in hip and lower limb surgery, and we do not recommend increasing the volume because wrong or nonstandard PENG block can elevate the risk of quadriceps femoris weakness, and a high volume will amplify this effect. ~~Therefore, the conventional volume is sufficient for PENG block.~~

**Response to key points in recommendation**

**Comment**

1. Consistent reporting of confidence intervals alongside P-values.

2. Softer language regarding type II error and the “optimal” dose conclusion.

3. Explicit note that secondary outcomes were exploratory and unadjusted for multiplicity.

**Response**: Thank you for your helpful and valuable comment. We have responded to these points in the major points and minor points above. Please check them
