# Supplementary material for: High-versus conventional-volume pericapsular nerve group (PENG) block for total hip arthroplasty: A randomized, controlled trial
Source: PLoS One. 2026 Apr 17;21(4):e0343615. doi: 10.1371/journal.pone.0343615 (PMC13089707; doi:10.1371/journal.pone.0343615)
Supplement: Attachment — Submitted filename: Response_to_Reviewers__auresp_3.docx [file pone.0343615.s006.docx]

Manuscript No.: PONE-D-25-27762R2

Title: High-versus conventional-volume pericapsular nerve group (PENG) block for total hip arthroplasty: A randomized, controlled trial

Dear Dr Nan Jiang,

Thank you very much for your letter and helpful advice. We have revised the manuscript and would like to re-submit it for your consideration. We would like to express our sincere thanks to you and the reviewers for your constructive and positive comments which help us promote our manuscript a lot.

We have addressed all the comments raised by the editor and reviewers, and the amendments are highlighted in red in the revised manuscript. Besides, we have adjusted some paragraphs.Also, we have listed a point-by-point response to the editor and reviewers' comments as follows.

Your consideration of our revised manuscript for publication in your journal will be highly appreciated!

We look forward to hearing from you soon.

Best regards,

Zhao-Hui Chen,

Department of Anesthesiology,

Third Affiliated Hospital of Chongqing Medical University ,

 No.1 Shuanghu Branch Road,

Chongqing, 401120, China;

Email: 650688@cqmu.edu.cn

**Response to the reviewer**

**Manuscript No.:** PONE-D-25-27762R2

**Title**: High-versus conventional-volume pericapsular nerve group (PENG) block for total hip arthroplasty: A randomized, controlled trial

**Comment 1:**

Type II Error: Language Remains Overconfident

Reviewer Concern (Persisting)

Despite prior feedback, the manuscript continues to assert that the likelihood of a type II error is “very low,” and uses this assertion to support conclusions of non-superiority. This is not statistically justified given:

• Small total sample size (n = 37)

• Power calculation limited to a single primary endpoint

• Absence of equivalence or non-inferiority design

Merely replacing “unlikely” with “very low” does not meaningfully soften the claim.

Why Further Softening Is Required

• A superiority trial that fails to detect a difference cannot rule out clinically meaningful effects.

• Power calculations do not eliminate type II error; they only constrain it under ideal assumptions.

• Repeated emphasis on “low type II error” risks misleading readers into inferring equivalence.

Required Change (Discussion)

Current text (representative):

“…our sample size has reached the pre-specified target, which reduces the probability of type II errors, which further supports that the postoperative analgesic effect of high-volume PENG block is not superior…”

Recommended replacement text:

“Although the study achieved the prespecified sample size for the primary endpoint, the relatively small cohort limits the precision of effect estimates. Accordingly, the possibility of a type II error cannot be fully excluded, and the absence of statistically significant differences should be interpreted with caution.”

**Response:** Thank you for your helpful and valuable comment. We have revised the description of type II errors and adopted the description of the reviewer（line292-294,line298-301）. Revision details were shown below:

~~Furthermore, our sample size has reached the pre-specified target, which reduces the probability of type II errors, which further supports that the postoperative analgesic effect of high-volume PENG block is not superior to that of the conventional volume group~~.(line292-294).

Although the study achieved the prespecified sample size for the primary endpoint, the relatively small cohort limits the precision of effect estimates. Accordingly, the possibility of a type II error cannot be fully excluded, and the absence of statistically significant differences should be interpreted with caution.(line298-301)

**Comment 2:**

Superiority vs. Equivalence: Inference Drift
Reviewer Concern
The trial is explicitly designed as a superiority trial, yet the Discussion and Conclusion increasingly imply equivalence or sufficiency, without appropriate methodological justification.
Why Further Softening Is Required
• Equivalence or non-inferiority cannot be inferred from a non-significant superiority test.
• Statements suggesting that conventional volume is “sufficient” or “optimal” exceed the evidentiary scope of the trial.
Required Change (Discussion)
Add the following clarifying sentence near the limitations paragraph:
“It should be noted that this study was designed as a superiority trial and was not powered or structured to formally assess equivalence or non-inferiority between treatment volumes.”

**Response:** Thank you very much for your helpful and valuable comment. We have deleted the description that conventional volume is sufficient(line323) , modified some sentences to soften the conclusion(line263-264,line267-268) and added the clarifying sentence near the limitations paragraph. (line301-302), revision details were shown below:

~~Therefore, the conventional volume is sufficient for PENG block in hip and lower limb surgery,~~

We have not found that the standard PENG block ~~did not~~ can block the peripheral branches of lumbar plexus even with high volume.

We used a higher volume, which may further ~~confirmed~~ strengthen that the high-volume PENG block did not enhance anesthetic or analgesic effects.

It should be noted that this study was designed as a superiority trial and was not powered or structured to formally assess equivalence or non-inferiority between treatment volumes.

**Comment 3:**

Secondary Outcomes and Multiplicity: Interpretation Still Too Strong

Reviewer Concern

While the authors now acknowledge that secondary outcomes were exploratory and unadjusted for multiplicity, interpretive language remains confirmatory.

Why Further Softening Is Required

• Multiple secondary endpoints increase false-negative and false-positive risk.

• Repeated-measures outcomes were analysed using independent comparisons, further limiting inference strength.

Required Change (Discussion)

Current text (representative):

“No significant differences were observed in dermatomal sensory block and motor block…”

Recommended replacement text:

“No statistically significant differences were observed in secondary outcomes; however, these analyses were exploratory, unadjusted for multiple comparisons, and may have been underpowered to detect modest or time-dependent effects.”

**Response**: Thank you for your helpful and valuable comment. We have revised the description of secondary outcomes and multiplicity. (line249-250,line315-318,line294-297), revision details were shown below:

~~Additionally, no significant differences were observed in dermatomal sensory block and motor block at all postoperative time points.~~ .(lne249-250)

~~Additionally, some secondary endpoints involved repeated measurement data; however, adjustment for multiple testing was not performed since these endpoints were exploratory. Fourthly, although the sample size was adequate for the primary endpoint, it was relatively small and may not have been sufficient to detect differences in the secondary endpoints.~~ (line315-318)

There were no differences in secondary outcomes such as sensory block and motor block; however, these analyses were exploratory, unadjusted for multiple comparisons, and may have been underpowered to detect modest or time-dependent effects.(line294-297)

**Comment 4:**

Conclusion: Practice Recommendations Are Premature

Reviewer Concern (Major)

The Conclusion currently makes clinical recommendations and suggests sufficiency of conventional volume, despite acknowledged limitations.

Why Further Softening Is Required

• PLOS ONE discourages definitive practice recommendations from small, single-centre trials.

• Conclusions should reflect what was tested, not what is implied.

Required Replacement Text: Conclusion (Full)

Current conclusion (summarized): “Therefore, the conventional volume is sufficient… we do not recommend increasing the volume…”

Recommended revised conclusion (replace entire section):

“In this randomized superiority trial, high-volume PENG block did not demonstrate superior postoperative analgesia compared with conventional-volume PENG block in patients undergoing primary total hip arthroplasty. While no statistically significant differences were detected in pain scores, motor function, or opioid-related outcomes, the study was not designed to establish equivalence between treatment volumes. Larger, adequately powered trials—ideally incorporating formal non-inferiority designs—are required to confirm these findings and to more precisely evaluate functional outcomes and uncommon adverse events.”

**Response**: Thank you for your helpful and valuable comment. We revised the conclusion and adopted the description of the reviewer(Line320-325,line326-332), revision details were shown below:

~~This study found that the high-volume PENG block achieved similar post-operative analgesic performance to the conventional-volume PENG block for primary THA. In addition, the incidence of quadriceps myasthenia of lower limbs was comparable to that of conventional PENG block.~~ ~~Therefore, the conventional volume is sufficient for PENG block in hip and lower limb surgery, and we do not recommend increasing the volume because wrong or nonstandard PENG block can elevate the risk of quadriceps femoris weakness, and a high volume will amplify this effect.~~

In this randomized superiority trial, high-volume PENG block did not demonstrate superior postoperative analgesia compared with conventional-volume PENG block in patients undergoing primary total hip arthroplasty. While no statistically significant differences were detected in pain scores, motor function, or opioid-related outcomes, the study was not designed to establish equivalence between treatment volumes. Larger, adequately powered trials—ideally incorporating formal non-inferiority designs—are required to confirm these findings and to more precisely evaluate functional outcomes and uncommon adverse events.
