# Supplementary material for: High-versus conventional-volume pericapsular nerve group (PENG) block for total hip arthroplasty: A randomized, controlled trial
Source: PLoS One. 2026 Apr 17;21(4):e0343615. doi: 10.1371/journal.pone.0343615 (PMC13089707; doi:10.1371/journal.pone.0343615)
Supplement: Attachment — Submitted filename: Response_to_Reviewers__auresp_4.docx [file pone.0343615.s007.docx]

Manuscript No.: PONE-D-25-27762R3

Title: High-versus conventional-volume pericapsular nerve group (PENG) block for total hip arthroplasty: A randomized, controlled trial

Dear Dr Nan Jiang,

Thank you very much for your letter and helpful advice. We have revised the manuscript and would like to re-submit it for your consideration. We would like to express our sincere thanks to you and the reviewers for your constructive and positive comments which help us promote our manuscript a lot.

We have addressed all the comments raised by the editor and reviewers, and the amendments are highlighted in red in the revised manuscript. We have listed a point-by-point response to the editor and reviewers' comments as follows.

Your consideration of our revised manuscript for publication in your journal will be highly appreciated!

We look forward to hearing from you soon.

Best regards,

Zhao-Hui Chen,

Department of Anesthesiology,

Third Affiliated Hospital of Chongqing Medical University ,

 No.1 Shuanghu Branch Road,

Chongqing, 401120, China;

Email: 650688@cqmu.edu.cn

**Response to the reviewer**

**Manuscript No.:** PONE-D-25-27762R3

**Title**: High-versus conventional-volume pericapsular nerve group (PENG) block for total hip arthroplasty: A randomized, controlled trial

**Comment 1:**

Discussion — Implied confirmation of “no effect”

Original sentence (lines 265–266)

“We used a higher volume, which may further strengthen that the high-volume PENG block did not enhance anesthetic or analgesic effects.”

“Further strengthen” implies confirmatory evidence of no effect, which is not justified in a superiority trial with limited power. This language subtly contradicts the carefully corrected type II error and non-equivalence statements later in the Discussion and Conclusion.

Therefore, I suggest to replace with “We used a higher volume; however, within the constraints of this superiority trial, no statistically significant enhancement of anesthetic or analgesic effects was detected.”

**Response:** Thank you for your helpful and valuable comment. We have revised the sentence（line267-270）. Revision details were shown below:

~~We used a higher volume, which may further strengthen that the high-volume PENG block did not enhance anesthetic or analgesic effects~~. We used a higher volume; however, within the constraints of this superiority trial, no statistically significant enhancement of anesthetic or analgesic effects was detected.

**Comment 2:**

Discussion — Overinterpretation of anatomical/mechanistic explanation

Original sentence (lines 261–262)

“We have not found that the standard PENG block can block the peripheral branches of lumbar plexus even with high volume.”

Reads as a general negative claim, rather than a study-limited observation. Risks being interpreted as a mechanistic conclusion rather than a contextual explanation.

Therefore, I suggest to replace with “In the present study, we did not observe evidence suggesting that increasing volume resulted in consistent blockade of peripheral branches of the lumbar plexus.”

**Response:** Thank you very much for your helpful and valuable comment. We have revised the description (line261-264), revision details were shown below:

~~We have not found that the standard PENG block can block the peripheral branches of lumbar plexus even with high volume~~. In the present study, we did not observe evidence suggesting that increasing volume resulted in consistent blockade of peripheral branches of the lumbar plexus.

**Comment 3:**

Discussion — Trend language bordering on inference

Original sentence (lines 283–284)

“Nevertheless, the conventional volume group exhibited a trend towards a delayed onset of first opioid use and a higher proportion of patients who remained opioid-free.”

“Exhibited a trend” can be read as directional inference, despite non-significance and lack of power. PLOS ONE generally prefers neutral phrasing when results are exploratory.

Therefore, I suggest to replace with “Nevertheless, numerically longer times to first opioid use and a higher proportion of opioid-free patients were observed in the conventional-volume group, although these differences were not statistically significant.”

**Response**: Thank you for your helpful and valuable comment. We have revised the description. (line285-290), revision details were shown below:

~~There was no statistically significant difference between the two groups concerning the time to first opioid use or the proportion of patients who did not require opioids within 48 hours post-operation. Nevertheless, the conventional volume group exhibited a trend towards a delayed onset of first opioid use and a higher proportion of patients who remained opioid-free~~. Nevertheless, numerically longer times to first opioid use and a higher proportion of opioid-free patients were observed in the conventional-volume group, although these differences were not statistically significant.

**Comment 4:**

Introduction — Strength of hypothesis framing (minor but relevant)
Original sentence (lines 105–106)
“We hypothesized that the dynamic pain score at 6 h after THA would be significantly lower in patients receiving the high-volume PENG block…”
While acceptable, the rest of the paper now strongly emphasises uncertainty, and this sentence slightly contrasts with the careful framing elsewhere. Not essential to change, but optional for consistency.

Therefore, it can be softened as “We hypothesized that the dynamic pain score at 6 h after THA might be lower in patients receiving the high-volume PENG block…”

**Response**: Thank you for your helpful and valuable comment. We revised the description (Line105-107), revision details were shown below:

We hypothesized that the dynamic pain score at 6 h after THA ~~would be significantly lower~~ might be lower in patients receiving the high-volume PENG block compared to those receiving the conventional-volume PENG block.
